# Assessment of chitosan addition to at-home bleaching gel with and without calcium on the physical properties of tooth enamel

**DOI:** 10.1590/1678-7765-2025-0513

**Published:** 2026-03-02

**Authors:** Laura Nobre Ferraz, Letícia de Souza Franco, Carolina Meneghin Barbosa, Marcos Roberto de Lima Benati, Tatiane Cristina Dotta, Waldemir Francisco Vieira-Junior, Débora Alves Nunes Leite Lima

**Affiliations:** 1 Fundação Hermínio Ometto - FHO Araras SP Brasil Fundação Hermínio Ometto - FHO, Araras, SP, Brasil; 2 Universidade de São Paulo Faculdade de Filosofia, Ciências e Letras Ribeirão Preto SP Brasil Universidade de São Paulo, Faculdade de Filosofia, Ciências e Letras, Ribeirão Preto, SP, Brasil; 3 Universidade de Campinas Faculdade de Odontologia de Piracicaba Departamento de Odontologia Restauradora Piracicaba SP Brasil Universidade de Campinas, Faculdade de Odontologia de Piracicaba, Departamento de Odontologia Restauradora, Piracicaba, SP, Brasil

**Keywords:** Chitosan, Calcium, Bleaching, Enamel microhardness, Surface roughness

## Abstract

Dental bleaching is a widely used esthetic treatment, but concerns persist regarding its potential effects on enamel integrity. The incorporation of remineralizing and protective agents into bleaching gels, such as calcium and chitosan, has been proposed to mitigate these effects. Objective: This *in vitro* study evaluated the effects of adding 2% chitosan to at-home bleaching gels with and without calcium on enamel physical properties. Methodology: Bovine enamel specimens stained with black tea were assigned to five groups (n = 12): control (no bleaching), 7.5% hydrogen peroxide (7.5% HP), 7.5% HP + 2% chitosan (7.5% HP+ Chi), 7.5% HP + calcium (7.5% HP + Ca), and 7.5% HP + calcium + chitosan (7.5% HP + Ca + Chi). Bleaching was performed for 14 days (1 hour/day). Color (∆L*, ∆a*, ∆b*, ∆Eab, ∆E_00_, and ∆WID), surface roughness, and surface microhardness (SMH) were evaluated at baseline (T1) and after treatment (T2). Mineral content (Ca, P, and the Ca/P ratio) was assessed by energy-dispersive X-ray spectroscopy and enamel morphology by scanning electron microscopy. Statistical analyses included the Kruskal-Wallis and Dunn's tests, mixed models, and ANOVA. Results: Groups containing chitosan (7.5% HP + Chi and 7.5% HP + Ca + Chi) showed the lowest surface roughness and the highest microhardness values. For these groups, SMH showed no decrease after treatment (T2), remaining similar to the initial values (T1). Color changes were similar among all bleached groups. Calcium, phosphorus, and Ca/P levels were the same for all groups. Conclusion: Adding 2% chitosan to 7.5% hydrogen peroxide-based at-home bleaching gels appears to be a promising approach to protect enamel against changes in roughness, microhardness, and mineral content without compromising bleaching efficacy.

## Introduction

Dental bleaching has become increasingly popular, but its safety remains a topic of debate. While some studies suggest minimal effects on enamel properties,^1^, ^2^ others report significant microstructural changes, such as morphological alterations in the enamel surface,^3^, ^4^, ^5^, ^6^ changes in the distribution of hydroxyapatite crystals, increased porosity,^7^ decreased surface and subsurface microhardness,^8^, ^9^ and greater adhesion of *Streptococcus mutans* to the enamel surface.^10^, ^11^ Furthermore, chemical studies of the enamel surface have shown alterations in the calcium/phosphate ratio and calcium loss suggesting that dental bleaching may structurally change the enamel, potentially influenced by factors such as pH, oxidative effects, or the composition of bleaching agents.^12^

To minimize enamel alterations following tooth bleaching, the incorporation of remineralizing agents into bleaching products has been proposed, including fluoride and calcium ions or hydroxyapatite.^13^, ^14^, ^15^, ^16^ Calcium supplementation in bleaching products aims to create a supersaturated environment with Ca^2+^ ions, preventing hydroxyapatite dissolution and promoting mineral redeposition into demineralized enamel.^17^ The mechanism involves the diffusion of calcium ions through the interprismatic spaces, enhancing remineralization by favoring crystal growth and stability of the enamel apatite lattice.^18^ The literature indicates that the presence of calcium in bleaching gels does not interfere with gel penetration or bleaching efficacy^14^ and reduces surface microhardness loss.^19^ However, their effect may be limited by short contact time and restricted diffusion of ions within the enamel structure.^17^

In this context, biopolymers have been investigated for their potential remineralizing and anti-erosive properties. Chitosan, a natural biopolymer derived from the deacetylation of chitin,^20^, ^21^ has recently been incorporated into bleaching gels.^21^, ^22^, ^23^, ^24^, ^25^ Several reasons explain why chitosan may be an appropriate additive to enhance bleaching gel formulations. These include its non-toxic, hydrophilic, biocompatible, biodegradable, and antibacterial characteristics, which make it a versatile material. Moreover, chitosan acts as a thickening and carrier agent due to its film-forming ability, creating a stable barrier over enamel or dentin that protects the tooth from demineralization and erosive challenges, maintaining the integrity and structure of dental and oral tissues.^26^ Previous studies have shown that incorporating chitosan reduces phosphorus loss and maintains the same bleaching efficacy as gels without chitosan.^22^ Furthermore, groups treated with chitosan-containing gels showed surface roughness and microhardness values resembling those of the untreated group.^23^

It is essential to evaluate the effects of remineralizing agents incorporated into bleaching gels, including the validation of bleaching efficacy and the prevention of undesirable side effects. Considering the significant action of chitosan, it is highly relevant to assess the effects of adding chitosan to bleaching gels with and without calcium. Since calcium is already present in some commercial formulations and has proven remineralizing benefits, understanding how chitosan interacts with calcium ions is essential to assess whether their combination provides additive or potentially synergistic effects in enhancing enamel protection during the bleaching process. Therefore, the aim of this in vitro study was to evaluate the effect of adding chitosan to commercial 7.5% hydrogen peroxide-based bleaching gels, with and without calcium, on bleaching efficacy, surface roughness, microhardness, and mineral content of enamel.

The null hypotheses tested were: 1) adding chitosan to bleaching gels, alone or in combination with calcium, does not affect bleaching efficacy; 2) the presence of chitosan in bleaching gels, alone or combined with calcium, has no effect on enamel roughness after tooth bleaching; 3) the presence of chitosan, alone or combined with calcium, does not affect enamel microhardness; and 4) the presence of chitosan, alone or combined with calcium, has no effect on enamel mineral content.

## Methodology

### Experimental design

A total of 120 bovine enamel specimens were stained with a black tea solution for six days and then randomly assigned to five experimental groups (n = 24 per group): Control (no bleaching), 7.5% hydrogen peroxide (7.5% HP), 7.5% hydrogen peroxide with 2% chitosan (7.5% HP + Chi), 7.5% hydrogen peroxide with calcium (7.5% HP + Ca), and 7.5% hydrogen peroxide with calcium and chitosan (7.5% HP + Ca + Chi). Table 1 summarizes the experimental groups and used materials.

**Table 1:** Experimental groups.

Group	Bleaching gel	Basic components	Calcium	Chitosan
Control	No bleaching	-	-	-
7.5% HP	Pola Day (SDI)	7.5% Hydrogen Peroxide, 47% Additives, 30% Glycerol, 20% Water, 0.1% Flavorings	No	No
7.5% HP+ Chi	Pola Day (SDI)	7.5% Hydrogen Peroxide, 47% Additives, 30% Glycerol, 20% Water, 0.1% Flavorings	No	2% chitosan
7.5% HP+ Ca	White Class 7.5% (FGM)	7.5% Hydrogen Peroxide, Neutralized Carbopol, Potassium Nitrate, Sodium Fluoride, Stabilizer, Humectant, Deionized Water	Calcium Gluconate	No
7.5% HP+ Ca + Chi	White Class 7.5% (FGM)	7.5% Hydrogen Peroxide, Neutralized Carbopol, Potassium Nitrate, Sodium Fluoride, Calcium Gluconate, Stabilizer, Humectant, Deionized Water	Calcium Gluconate	2% chitosan

Each group was divided into two subgroups (n=12) according to the performed analyses. Sample size calculation, based on a pilot study with three specimens per group (comparing the non-bleached group with the 7.5% HP + Ca + Chi group), indicated that 11 specimens per group would yield 90% power (β=0.9) at α = 0.01. Therefore, the sample size was rounded up to 12 specimens per group. One subgroup was used for color, surface roughness, energy-dispersive X-ray spectroscopy (EDS), and scanning electron microscopy (SEM) analyses, whereas the other was used exclusively for surface microhardness (SMH) evaluation.

The following parameters were assessed: color changes (∆L*, ∆a*, ∆b*, ∆E*_ab_, ∆E_00_), surface roughness (Ra), surface microhardness (SMH), elemental composition (Ca, P, and Ca/P ratio quantification), and surface morphology by SEM. Color, roughness, and microhardness were evaluated at baseline (T1) and after treatment (T2, 14 days), whereas EDS and SEM analyses were conducted only at T2.

**Figure 1:**
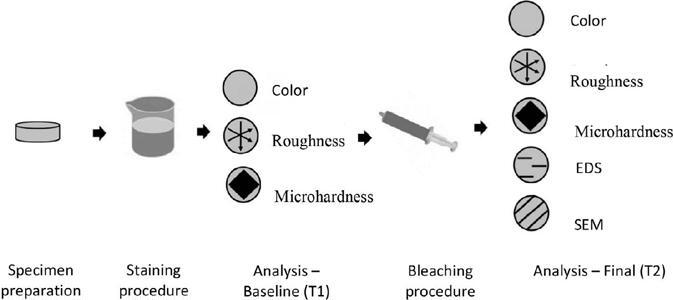
Experimental flowchart of the study

To provide a clear overview of the experimental workflow, Figure 1 illustrates the experimental flowchart.

### Specimen preparation

The specimens in this study were bovine teeth. After collection, they were cleaned with a paste made of pumice stone and water using a rubber cup. The bovine teeth were immersed in distilled water until use. The crown was separated from the root using a diamond disc (KG Sorensen, Barueri, SP, Brazil). Cylindrical fragments with a diameter of 7 mm were obtained using a 10 mm diamond-coated saw drill (Rocast Perfection, Amatools; Piracicaba, SP, Brazil) on a bench drill. The enamel and dentin were leveled with silicon carbide sandpapers (#600 and #1200 grits) on a rotary polishing machine (Aropol E, Arotec, Cotia; SP, Brazil). The enamel polishing was performed with felt (TCT, TWI – Arotec, Cotia; SP, Brazil) and diamond pastes (1 and ¼ μm). At the end of this process, the specimens had a thickness of 2.0 mm (1 mm of enamel and 1 mm of dentin). To standardize the position of the specimens during color and roughness analyses, a spherical diamond tip #1011 was used to mark the lateral side of each specimen.

### Staining procedure

A black tea solution was used to stain the specimens. The solution was prepared by boiling 100 mL of distilled water for 5 minutes. Then, 1.6 g of black tea (Leão Junior S.A., Curitiba, PR, Brazil) was infused in the hot water for 5 minutes. The specimens were immersed in the staining solution without agitation at 37°C, which was replaced every 24 hours for six days 27. The solution was neutralized using sodium hydroxide to prevent specimen demineralization since black tea has an acidic pH. After six days of staining, the specimens were stored in artificial saliva for two weeks to stabilize their color. Once the specimens were prepared, the groups were randomized. For the specimens designated for color, surface roughness, EDS, and SEM analysis, randomization was based on the L* value, excluding extreme values to reduce initial variability between groups.^3^ For the other specimens, randomization was done according to surface microhardness analysis, excluding extreme values to also minimize initial variability between groups.

The specimens were kept in artificial saliva throughout the experiment. Artificial saliva was used for specimen storage to reproduce as closely as possible the conditions in the oral cavity. In the mouth, teeth are constantly exposed to saliva, which plays a crucial role in maintaining mineral balance by the continuous processes of demineralization and remineralization. Therefore, immersing the specimens in artificial saliva more realistically simulates the intraoral environment. The absence of this medium could lead to mineral changes that fail to accurately reflect the clinical situation. This approach is consistent with previous studies and represents a widely accepted procedure in in-vitro research involving enamel demineralization and remineralization. The artificial saliva was prepared with the following formulation: 1.5 mM Ca, 0.9 mM PO4, and 150 mM KCl in a 20 mM Tris buffer solution, pH 7.0.^28^

### Teeth bleaching procedure

The specimens were bleached over 14 days. When not undergoing bleaching, the specimen were stored in artificial saliva at 37°C. For the groups containing chitosan, this substance was weighed on an analytical balance according to the desired concentration and immediately incorporated into the bleaching gel prior to application. Chitosan was manually blended with the bleaching gel for 20 seconds using a spatula to ensure complete incorporation. To perform the treatment, the specimens were placed on a glass plate. A 1 mm-thick layer of bleaching gel was uniformly applied over each specimen to ensure standardization of the amount of used material. For all groups, the bleaching gels were applied once daily for 1 hour. During the application time, the specimens were kept in an incubator at 37°C. After the treatment, the bleaching gel was removed from the specimen surface using cotton, followed by rinsing the surface with distilled water. Subsequently, the specimens were stored again in artificial saliva at 37°C.

### Color analysis

Color analysis was performed after staining (baseline, T1) and 24 hours after the final bleaching session (T2). A sample holder (Teflon device) was used to position the specimens inside a light chamber (GTI Mini Matcher MM1e, GTI Graphic Technology Inc., Newburgh, NY, USA), in which the use of daylight was standardized. A spectrophotometer was positioned in a sample holder so that it was perpendicular to the specimen surface. The Konica Minolta CM-700d spectrophotometer was calibrated, and three readings were taken for each specimen. For the first reading, the specimen was positioned with the marking facing the operator. Then, the specimen was rotated 45° and a new reading was performed; then, it was rotated another 45° and another reading was taken. The average of the values was calculated to obtain a single value per specimen. The obtained values were quantified for the L*, a*, and b* coordinates. The difference between the initial and final readings of L*, a*, and b* coordinates was calculated to determine ΔL, Δa, and Δb values. The WID was calculated using the formula WID=0.511L – 2.324a – 1.100b*, and the ΔWID was determined as the difference between the final and baseline values (ΔWID = WID final − WID baseline).^29^ Color change was calculated using the equation ΔE*_ab_ = [(L1 − L0)^2^ + (a1 − a0)^2^ + (b1 − b0)^2^]½.^3^ Additionally, ΔE_00_ was calculated using the following equation:

$$\Delta E_{00}={\left[{\left(\frac{\Delta L^{\prime }}{K_{L}S_{L}}\right)}^{2}+{\left(\frac{\Delta C^{\prime }}{K_{C}S_{C}}\right)}^{2}+{\left(\frac{\Delta H^{\prime }}{K_{H}S_{H}}\right)}^{2}+R_{T}\left(\frac{\Delta C^{\prime }}{K_{C}S_{C}}\right)\left(\frac{\Delta H^{\prime }}{K_{H}S_{H}}\right)\right]}^{1/2}$$

### Surface roughness

Surface roughness analysis was performed after staining (baseline, T1) and 24 hours after the final bleaching session (T2) using a profilometer (Surf-Corder 1700, Kosaka, Tokyo, Japan). The specimens were fixed on an acrylic stub, with markings indicating the appropriate reading angles. For the first reading, the specimens was positioned with the marking facing the operator. Then, the specimens was rotated 45° and a new reading was performed; then, it was rotated another 45° and another reading was taken. The average of the values was calculated to obtain a single value per specimens. The profilometer was programmed to perform readings with a measurement length of 1.25 mm, a speed of 0.1 mm/s, and a cut-off of 0.25 mm.

### Surface microhardness

Surface microhardness analysis was performed after staining (baseline, T1) and 24 hours after the final bleaching session (T2). To avoid interference with the light reflectance pattern due to indentations potentially altering the surface topography of the specimen, specimens were exclusively prepared for the microhardness test. The microhardness values were obtained by calculating the arithmetic mean of five indentations in the central area of each specimen, spaced 100 μm apart. A Knoop microhardness tester (HMV-2000, Shimadzu, Tokyo, Japan) with a 50-g load was used for 5 seconds.^30^

### Energy dispersive x-ray spectroscopy

This analysis was conducted after all other analyses in this study had been completed. Overall, five specimens from each group were randomly selected.^4^ The analysis was conducted using the Vantage microscope (Acquisition Engine Company, Tokyo) with the Easymicro Voyager digital software, version 5.2. The surface of the specimens was prepared by applying a carbon coating. Calcium and phosphorus content were quantified in five regions of each specimen using a scanning electron microscope (SEM, Jeol JSM5600LV, Tokyo, Japan), with typical energy settings of 15 kV, magnification of 100×, and a PHA deadtime ranging from 20 to 25%. The average values of the chemical elements were calculated, and a single value per specimens was recorded.^4^

### Scanning electron microscopy

This analysis was conducted after all other analyses in this study had been completed. Overall, two specimens from each group were selected for scanning electron microscopy (JSM-5600LV, JEOL, Tokyo, Japan). The specimens were prepared by dehydration in ethanol at concentrations of 50, 60, 70, 80, 90, and 100%. Each solution was used for 20 minutes, except for the 100% ethanol solution, which was applied for 60 minutes. After dehydration, the specimens were dried at room temperature for 12 hours. Subsequently, the specimens were mounted on acrylic stubs, and a thin gold layer was deposited on the surface using a cathodic sputter coater (SCD 050, Balzers Union Aktiengesellschaft, Balzers, Liechtenstein). Representative images of each surface were captured at a magnification of 4000×.

### Statistical analysis

Statistical analyses were on R under a 5% significance level. Initially, descriptive and exploratory data analyses were performed. A mixed linear model for repeated measures over time was applied to analyze Knoop microhardness. As the roughness data showed asymmetric distribution, they were analyzed using a generalized mixed linear model for repeated measures over time. Color data (∆L*, ∆a*, ∆b*, ∆E_ab_, ∆E_00_) were analyzed using the non-parametric Kruskal-Wallis and Dunn's tests and one-way ANOVA with Tukey's post-hoc test (∆WID). EDS analysis data were subjected to one-way analysis of variance (ANOVA) to compare the groups.

## Results

### Color analysis

The results of the color analysis are shown in Table 2.

**Table 2:** Mean (standard deviation) of color variation variables measured by reflectance spectrophotometry across different groups.

Group	∆L*	∆a*	∆b*	∆E_ab_	∆E_00_	∆WID
No bleaching	−1.10 (0.63)^A^	0.20 (0.27)^A^	4.98 (5.76)^A^	1.56 (0.77)^A^	1.77 (0.79)^A^	−3.18 (8.68)^A^
7.5% HP	6.46 (1.93)^B^	−0.78 (0.77)^B^	−22.78 (5.07)^B^	25.46 (5.16)B	13.04 (4.00)^B^	28.52 (29.23)^B^
7.5% HP + Chi	6.84 (1.96)^B^	−0.64 (0.58)^B^	−24.03 (5.37)^B^	27.25 (6.37)B	13.08 (4.25)^B^	40.15 (17.54)^B^
7.5% HP + Ca	6.08 (1.57)^B^	−0.75 (0.88)^B^	−22.09 (5.14)^B^	24.88 (4.10)B	13.46 (4.56)^B^	32.69 (26.88)^B^
7.5% HP + Ca + Chi	6.05 (2.28)^B^	−0.60 (0.84)^B^	−25.53 (5.51)^B^	24.61 (6.27)B	14.22 (4.92)^B^	37.15 (27.42)^B^
p-value	<0.0001	0.0019	<0.0001	<0.0001	<0.0001	<0.0001

Distinct letters (comparing the groups in each variable) indicate statistically significant differences (p≤0.05).

The findings indicate that all bleached groups showed an increase in ∆L values and a decrease in ∆a and ∆b values. Regarding total color variation, the ∆E_ab_ and ∆E_00_ values were significantly lower in the non-bleached group than in all other groups (p<0.05).

Furthermore, across all color analysis parameters in this study, no significant differences were observed among the bleached groups. However, all bleached groups showed statistically significant differences from the non-bleached group (p<0.0001).

### Surface roughness

The results of the surface roughness analysis are shown in Table 3.

**Table 3:** Mean (standard deviation) of surface roughness (Ra) as a function of group and time.

Group	Time	
	Initial	Final
	Mean (standard deviation)	Mean (standard deviation)
No bleaching	0.07 (0.01)^Aa^	0.07 (0.01)^Ac^
7.5% HP	0.07 (0.01)^Ba^	0.13 (0.01)^Aa^
7.5% HP + Chi	0.07 (0.01)^Ba^	0.10 (0.01)^Ab^
7.5% HP + Ca	0.07 (0.01)^Ba^	0.13 (0.01)^Aa^
7.5% HP + Ca + Chi	0.07 (0.01)^Ba^	0.10 (0.01)^Ab^

Distinct letters (uppercase horizontally and lowercase vertically) indicate statistically significant differences (p≤0.05). p(group<0.0001; p(time)<0.0001; p(interaction)<0.0001.

When comparing the initial and final time points, all bleached groups showed statistically significant differences (p<0.0001), with surface roughness values increasing at the final time point. The control group was the only group that showed no statistically significant difference between the initial and final measurements.

At the initial time point, no statistically significant differences were observed among the groups. However, at the final time point, the control group showed the lowest roughness values, which differed statistically from all bleached groups (p<0.0001). Among the bleached groups, the 7.5% HP and 7.5% HP + Ca groups showed the highest roughness values, with no significant differences between each other. In contrast, the 7.5% HP + Chi and 7.5% HP + Ca + Chi groups showed the lowest roughness values, which also failed to significantly differ from one another.

### Surface microhardness

The results of the surface microhardness analysis are shown in Table 4.

**Table 4:** Mean (standard deviation) of superficial Knoop microhardness (KHN) as a function of group and time.

Group	Time	
	Initial	Final
	Mean (standard deviation)	Mean (standard deviation)
No bleaching	284.44 (7.47)^Aa^	284.61 (9.06)^Aa^
7.5% HP	280.55 (7.59)^Aa^	254.49 (6.27)^Bb^
7.5% HP + Chi	282.02 (7.72)^Aa^	284.46 (5.55)^Aa^
7.5% HP + Ca	282.00 (7.48)^Aa^	251.44 (6.98)^Bb^
7.5% HP + Ca + Chi	280.53 (6.17)^Aa^	282.48 (7.84)^Aa^

Distinct letters (uppercase horizontally and lowercase vertically) indicate statistically significant differences (p≤0.05). p(group<0.0001; p(time)<0.0001; p(interaction)<0.0001.

When comparing the initial and final time points, the 7.5% HP, 7.5% HP + Ca groups showed statistically significant differences (p<0.0001), with lower microhardness values at the final time point than at the initial one. In contrast, the control group, 7.5% HP + Chi, and 7.5% HP + Ca + Chi groups showed no statistically significant differences between the initial and final measurements.

At the initial time point, no statistically significant differences were observed between the groups. However, at the final time point, the 7.5% HP + Chi and 7.5% HP + Ca + Chi groups showed the highest microhardness values, which were statistically insignificant from those of the control group. On the other hand, the 7.5% HP and 7.5% HP + Ca groups showed the lowest microhardness values between all groups, no significant differences from each other, and statistically differences from the control and the other bleached groups (p<0.0001).

### Energy dispersive x-ray spectroscopy (EDS)

The data from the calcium quantification analysis are shown in Table 5. No statistically significant differences were observed between the groups regarding calcium content (p>0.05). However, the p-value was close to the significance threshold (p=0.0646) and the effect size of the group on calcium quantity was large (f=1.12), suggesting that further studies with larger sample sizes are necessary.

**Table 5:** Mean (standard deviation) for elements calcium, phosphorus, and calcium/phosphorus values according to groups.

Group	Calcium (Ca)	Phosphorus (P)	Ca/P
No bleaching	71.45 (0.15)^a^	28.56 (0.16)^a^	2.50 (0.02)^a^
7.5% HP	71.14 (0.08)^a^	28.86 (0.08)^a^	2.46 (0.01)^a^
7.5% HP + Chi	71.06 (0.23)^a^	28.94 (0.23)^a^	2.46 (0.03)^a^
7.5% HP + Ca	71.18 (0.27)^a^	28.82 (0.27)^a^	2.47 (0.03)^a^
7.5% HP + Ca + Chi	71.46 (0.06)^a^	28.54 (0.06)^a^	2.50 (0.01)^a^
p-value	p=0.0646	p=0.0725	p=0.0681

Effect size of calcium (f)=1.12. Effect size of phosphorus (f)=1.10 Effect size of calcium/phosphorus (f)=1.11 (large effect according to Cohen 1988 and 1992). Same letters indicate no statistically significant differences (p>0.05).

Similarly, no statistically significant differences were found between the groups regarding phosphorus content (p>0.05), as in Table 5. However, the p-value was also close to the threshold (p=0.0725), and the effect size for phosphorus quantification was large (f=1.10), reinforcing the need for additional studies with larger specimens.

The data on the calcium-to-phosphorus ratio are available in Table 5. Similar trends were observed for this ratio as in the calcium and phosphorus quantifications, with no significant differences between groups. However, the p-value remained close to the threshold (p=0.0681) and the effect size was large (f=1.11), further emphasizing the need for future investigations with expanded sample sizes.

### Scanning electron microscopy

**Figure 2:**
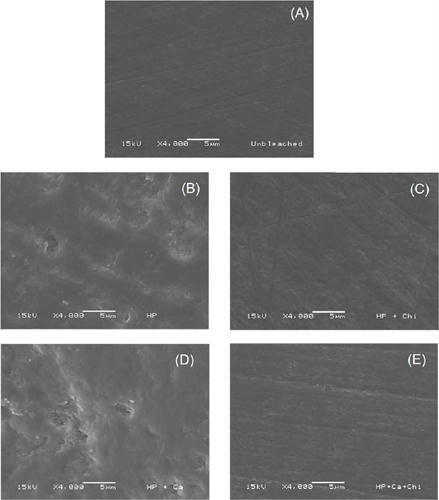
Representative scanning electron microscopy images (4000*) of the specimens according to the group: (A) without bleaching, (B) 7.5% HP; (C) 7.5% HP + Chi; (D) 7.5% HP + Ca and (E) 7.5% HP + Ca + Chi.

SEM images are shown in Figure 2. The non-bleached group showed a smooth and uniform enamel surface. In contrast, all bleached groups showed surface alterations, with varying degrees of porosity and depressions depending on the bleaching gel.

The 7.5% HP and 7.5% HP + Ca groups showed highly similar surface characteristics, with a pronounced roughness pattern and deeper demineralization areas than the groups containing chitosan. Although the chitosan-treated groups also showed enamel alterations, the irregularities emerged more evenly distributed across the surface. However, these changes seemed to be more superficial, with shallower alterations and no distinct areas of pronounced demineralization.

## Discussion

This study evaluated adding 2% chitosan to 7.5% hydrogen peroxide-based bleaching gels with or without calcium and its effects on the physical properties of bleached enamel. Bleaching gels without chitosan served as active controls for their respective chitosan-containing formulations, enabling the isolated assessment of the 2% chitosan effect (w/w). It is important to highlight that only one hydrogen peroxide concentration was tested in this study: the highest level typically in at-home bleaching gels, which was selected to simulate a worst-case scenario regarding potential enamel alterations. Previous studies have shown that the extent of adverse effects on dental tissues caused by tooth bleaching is strongly influenced by the concentration, pH, and chemical composition of the bleaching agent, as well as by its contact time with the tooth surface.^31^, ^32^, ^33^, ^34^, ^35^, ^36^ Therefore, differences in peroxide levels may influence the kinetics of oxygen release, enamel permeability, and consequently, the extent of surface changes, prohibiting the direct extrapolation of the results to products containing lower or higher peroxide concentrations. Future studies including different peroxide concentrations are necessary to confirm whether the protective effects in this study are consistent across other formulations.

In this study, tooth color measurements were performed using the Konica Minolta spectrophotometer, which provides objective and reproducible values. Previous studies have also used the EasyShade System to assess bleaching outcomes.^37^, ^38^, ^39^, ^40^ While both devices offer reliable color evaluation, some differences in readings have been reported, likely due to variations in light source, calibration, and measurement geometry.^41^, ^42^ Studies comparing EasyShade with Minolta have generally found good agreement between the two systems, the EasyShade may slightly overestimate or underestimate certain color parameters when compared to Minolta.^43^ Therefore, interpreting color changes should consider the used device as small differences may arise between instruments even under standardized conditions.

The first hypothesis of this study was accepted, as the addition of 2% chitosan to the bleaching gel, with or without calcium, did not interfere with enamel bleaching efficacy. No statistically significant differences were found between the bleached groups, whereas all bleached groups differed from the control group in all color analyses. The results indicate that tooth bleaching was effective across all groups as they increased L values* and decreased b values*, leading to a lighter and less yellowish tooth color.^44^, ^45^ Furthermore, when the overall color change was evaluated using ∆E_ab_ and ∆E_00_, the results exceeded the thresholds for perceptibility and acceptability. Specifically, ΔE_ab_ values were higher than 1.2 for perceptibility and greater than 2.7 for acceptability. Similarly, ΔE_00_ values exceeded 0.8 for perceptibility and 1.8 for acceptability.^46^ For ΔWID, the values were also higher than 0.72 for perceptibility and 2.60 for acceptability.^47^ These findings are in line with previous studies showing that adding chitosan to 6% hydrogen peroxide-based bleaching gels^22^ or to 35% hydrogen peroxide-based gels with or without calcium^23^ failed to negatively affect bleaching efficacy.

The surface roughness results showed that all bleached groups obtained an increase in roughness values when comparing the initial and final time points, which is likely associated with the action of hydrogen peroxide in the bleaching gel formulations. The effect of hydrogen peroxide on the entire dental structure is unavoidable as oxygen radicals interact nonspecifically, meaning that they affect the entire dental structure rather than exclusively targeting pigment molecules.^48^ As a result, hydrogen peroxide can lead to the mineral dissolution of enamel,^4^, ^15^ which explains the observed increase in surface roughness in the bleached groups. This finding is further supported by SEM images as all bleached groups (Figures 2B, 2C, 2D, and 2E) showed surface characteristics distinct from the non-bleached group (Figure 2A), evincing demineralization patterns that varied according to the bleaching gel used.

Despite the increase in roughness in the bleached groups, those containing chitosan showed lower roughness values than their respective commercial controls. This trend is also evident in the SEM images, in which the chitosan-containing groups (Figures 2C and 2E) showed a more uniform surface with less demineralization than the bleached groups without chitosan (Figures 2B and 2D). The second null hypothesis in this study was rejected based on these findings. Although all bleached groups differed from the control group at the final time point, adding chitosan to the bleaching gel with or without calcium led to a smaller increase in enamel surface roughness.

This study also evaluated the effects of bleaching on enamel microhardness. The current literature reports that bleaching agents may have little to no effect on enamel microhardness.^49^, ^50^ According to ISO 28399,^51^ a variation of up to 10% is considered acceptable for external tooth bleaching products. In this study, some groups showed changes approaching this 10% threshold. However, certain experimental groups, particularly those containing chitosan, showed minimal or nearly no reduction in surface microhardness. These findings highlight that, although the observed changes stayed within the limits established by ISO standards, it is desirable to explore bleaching protocols or adjunctive treatments that minimize deleterious effects on enamel, ideally achieving zero or near-zero alterations in microhardness.

The effect of chitosan was also notable in the surface microhardness analysis. The initial and final microhardness values remained unchanged in the chitosan-containing bleached groups, showing a behavior resembling that of the non-bleached group. Additionally, at the final time point, the chitosan-containing groups differed significantly from their respective commercial bleaching gel controls but showed no statistical difference from the non-bleached group. This result rejects the third null hypothesis in this study. Furthermore, the SEM images of the chitosan-containing bleached groups (Figures 2C and 2E) evinced that, despite some surface alterations, these changes seem superficial, and their enamel surfaces resemble those of the non-bleached group (Figure 2A).

Chitosan can electrostatically bind to negatively charged surfaces, such as dental enamel, forming multilayers.^52^, ^53^ These chitosan multilayers remain stable in acidic environments, such as the low pH of bleaching gels.^54^, ^55^ Consequently, chitosan layers can inhibit mineral loss, reducing, for example, phosphate ion depletion, which typically occurs during tooth bleaching procedures.^54^, ^55^ Additionally, the multilayers formed by chitosan may have enhanced the mineral recovery of enamel via artificial saliva. It has been suggested that chitosan adsorption onto the artificial saliva layer can protect hydroxyapatite crystals by forming cross-links between the enamel surface and saliva.^56^ Thus, the chitosan layer may have facilitated the incorporation of calcium and phosphate minerals from artificial saliva into the enamel, contributing to the positive results in this study.

The effect of calcium on enamel during tooth bleaching was also evaluated in this study. Adding calcium can saturate the bleaching gel, facilitating its incorporation into the hydroxyapatite crystals of the enamel.^19^ This has been associated with an increased resistance of these crystals to demineralization and a reduction in the harmful effects caused by hydrogen peroxide, such as less surface microhardness loss and decreased mineral loss.^15^, ^23^ In this study, however, calcium was unable to reduce the adverse effects of bleaching. In the surface roughness and in surface microhardness analyses, the group treated with 7.5% HP + Ca failed to differ from the 7.5% HP group. This result is further supported by SEM images, in which the 7.5% HP + Ca group (Figure 2C) showed a surface that resembled that of the 7.5% HP group (Figure 2B) more than that of the non-bleached or chitosan-containing groups. The calcium-containing bleaching gel only performed well in the 7.5% HP + Ca + Chitosan group, indicating that chitosan played a significant role. However, the results for this group failed to statistically differ from those of the 7.5% HP + Chitosan group, suggesting that the action of calcium was either very low or nonexistent.

This result aligns with previous studies indicating that calcium added to bleaching gels failed to positively affect surface microhardness or surface roughness after bleaching.^57^ Sasaki, et al.^57^ (2015) suggest that the increase in roughness may be associated with calcium deposition on the enamel surface. Enamel demineralization caused by hydrogen peroxide gels disrupts the ionic balance, facilitating the deposition of calcium on the enamel surface.^58^ Additionally, the literature shows that the calcium in the bleaching gel is unable to reduce the permeability of the dental structure, and it is suggested that this may be linked to the extended application time of at-home bleaching products.^59^ This factor may also contribute to the results in this study.

This study also quantified calcium, phosphorus, and the calcium-to-phosphorus ratio in the enamel. The levels of calcium and phosphorus in enamel indicate the demineralization and remineralization processes.^60^ Loss of calcium and/or phosphorus can occur during bleaching due to the dissolution of hydroxyapatite crystals.^60^ The inclusion of EDS data was particularly valuable for supporting and confirming the results obtained from the microhardness and surface roughness analyses. While surface roughness provides indirect evidence of enamel alterations, EDS directly quantifies the elemental changes associated with demineralization or remineralization, enabling a more comprehensive interpretation of these phenomena. Moreover, the use of EDS as a complementary tool is well established in the literature, and several studies investigating enamel or dentin demineralization have combined EDS with microhardness and/or roughness analyses to strengthen their conclusions.^1^, ^4^, ^61^ Importantly, to our knowledge, no previous studies have evaluated the effect of adding chitosan to desensitizing agents prior to dental bleaching. Considering the interactions between chitosan and dental surfaces, the EDS analysis was essential to quantitatively assess whether this component could influence the mineral gain or loss of enamel after bleaching.

No statistically significant differences were found in EDS analysis between all the evaluated groups, leading to the acceptance of the fourth null hypothesis in this study. The bleached group with 7.5% HP failed to differ from the non-bleached group or the other groups treated with gels containing calcium or chitosan. This result contrasts with some studies that have shown that hydrogen peroxide reduces the amount of calcium in enamel.^61^, ^62^ However, Tezel, et al.^31^ (2007) has shown that mineral loss in enamel was greater with 35% and 38% hydrogen peroxide than with 10% carbamide peroxide. Therefore, it is likely that mineral loss is directly related to the concentration of hydrogen peroxide, which supports the findings of this study, as all the used bleaching gels contained low concentrations of hydrogen peroxide. Furthermore, the specimens remained immersed in artificial saliva throughout the experiment, which may have facilitated the remineralization of the whitened enamel.^60^

Still regarding the results of the EDS analysis, it is also important to discuss effect sizes. The effect sizes reported for Ca (f=1.12), P (f=1.10), and the Ca/P ratio (f=1.11) were calculated using Cohen's f for ANOVA based on the observed group means and standard deviations. Cohen's f is calculated as the square root of the ratio between the variance explained by the group factor and the residual variance. According to conventional thresholds (small: f=0.10; mediumml: f=0.25; large: f=0.40), these values indicate a large effect, which generally means that differences between groups are substantial to the variability within groups. However, in this study, no statistically significant differences were observed between groups for the EDS analyses. Reporting the effect sizes provides additional context, enabling readers to understand the magnitude of potential differences. In this case, it confirms that, despite the calculation of large effect sizes, the treatments failed to meaningfully alter the enamel mineral content, reinforcing the conclusion that the evaluated interventions minimally impacted the elemental composition of enamel. Nevertheless, further studies with a larger sample size are warranted to confirm these findings and provide more robust evidence regarding the potential effects of the treatments on enamel mineral content.

The results in this study provided valuable evidence regarding the behavior and interaction of the tested materials under bleaching conditions, highlighting their potential relevance for future dental applications. Nonetheless, certain limitations should be acknowledged. The in vitro nature of the experiment fails to fully replicate the complex biological environment of the oral cavity, including salivary dynamics, enzymatic activity, and mechanical forces. Moreover, the limited number of specimens used for EDS and SEM analyses may restrict the generalization of these specific observations. The use of artificial saliva as a storage medium represents another simplification of the intraoral environment. Finally, the absence of clinical validation prevents direct extrapolation of these findings to real clinical scenarios. Further in situ and in vivo studies, including long-term assessments with larger sample sizes, are warranted to confirm these preliminary results and support the translation of these materials into clinical practice.

## Conclusion

Incorporating 2% chitosan into the 7.5% hydrogen peroxide bleaching gel seems a promising strategy to minimize the negative effects of bleaching, preserving surface roughness, microhardness, and enamel mineral content without compromising bleaching efficacy.

## Data Availability

All data generated and analyzed in this study are included in this published article.
